# Clinical Trials in Chronic Arthritic Diseases with Underestimated Impact of Placebo Effects on Study Size Calculation

**DOI:** 10.3390/jcm12020429

**Published:** 2023-01-05

**Authors:** Katharina Richetti, Jennifer Gebetsberger, Werner Streif, Michael Schirmer

**Affiliations:** 1 Department of Internal Medicine, Clinic II, Medical University of Innsbruck, 6020 Innsbruck, Austria; 2 Department of Pediatrics, Clinic I, Medical University of Innsbruck, 6020 Innsbruck, Austria

**Keywords:** placebo, drug development, pharmacology, clinical trials, rheumatology, trial size

## Abstract

Whether and to which extent placebo treatment in double-blinded randomized controlled clinical trials is effective in chronic arthritic diseases has not been studied before. Therefore, a systematic literature search was undertaken to detect eligible trials. Demographic data of the placebo groups as well as concomitant and previous disease outcomes were collected. Analyses of significant bivariate correlations and linear regression between clinical endpoints and characteristics of the placebo groups were performed. A total of 152 double-blinded randomized controlled studies, including 21,616 participants in the placebo groups, was analyzed. The results of bivariate correlations and linear regressions revealed significant positive associations between responses in the placebo groups and the following factors: (i) naïvety of previous treatment and (ii) early stage of disease. In addition to the clinical relevance, the results support the importance of the placebo effect on study size calculations, and will allow an optimized calculation of patients’ numbers for early placebo-controlled trials conducted in patients with chronic arthritic diseases.

## 1. Introduction

The debate concerning whether placebo treatment works for medical conditions has continued since Beecher’s landmark paper “The Powerful Placebo” published in 1955 [1]. For the regular clinical setting, consensus of experts has been reached in that placebo effects should be considered as part of the treatment and maximized to improve treatment outcomes [2].

On the other side, placebos are used in clinical trials which are designed to assess possible clinical effects of new treatment approaches when compared to placebos. Placebo-controlled double-blinded and randomized trials are considered as gold standard to achieve a high level of evidence for the comparison of new treatments with placebos [3]. However, patients receiving placebos often experience symptomatic improvement, particularly for subjective rather than objective outcomes [4]. Thus it is not surprising that clinical trials, such as for major depressive disorders, are often confronted with a high variability of placebo response, which complicates the interpretation of clinical outcomes. As a consequence, approximately half of the trials of newer marketed antidepressants in the US Food and Drug Administration (FDA) database failed to demonstrate superiority over placebo [5].

However, such variations of placebo responses also occur in other chronic diseases. For inflammatory bowel disease, for example, the placebo response rates of clinical improvement and remission rates vary between 5 and 50% in Crohn’s disease and between 10 and 35% in ulcerative colitis [6]. Interestingly, comparable to the placebo effects in trials with neuropsychiatric disorders, there has been a rise of the placebo response in trials with rheumatoid arthritis (RA) over the last two decades [7]. In 32 selected placebo-controlled trials on the effects of biological and targeted synthetic disease-modifying agents, an increase in placebo ACR50 and ACR70 responses was reported, which remained significant after controlling for potential confounders. The authors explained this effect with possible shifting of the RA phenotype, changes in trial design, and expectation bias. As a matter of fact, outcome scores of rheumatic diseases including RA, psoriatic arthritis (PsA) and axial spondyloarthritis (SpA) include subjective assessments of disease activity.

Thus, placebo effects may vary not only in neuropsychiatric but also in immune-mediated diseases. The aim of this meta-analysis is to further analyze the effects of placebos on disease activities in clinical trials with RA, PsA, and SpA, and to examine the placebo effects on power calculations of clinical trials in the most prominent rheumatic diseases.

## 2. Literature Search and Methods

### 2.1. Used Guidelines

Study selection, assessment of eligibility criteria, data extraction, and statistical analysis were performed in accordance with the methodology guidelines from Cochrane [8]. The findings are reported according to the Preferred Reporting Items for Systematic Reviews and Meta-Analysis (PRISMA) statement [9].

### 2.2. Data Sources and Searches

To identify all relevant publications about the placebo effect in chronic arthritic diseases, a systematic literature search was performed in the bibliographic databases Medline (PubMed) and The Cochrane Library (via Wiley). Double-blind placebo-controlled randomized clinical trials, clinical trials, evaluation studies, and validation studies conducted in rheumatoid arthritis (RA), axial spondyloarthritis (SpA), and psoriatic arthritis (PsA) were searched. Juvenile rheumatoid arthritis (jRA) was not included in this review, to avoid an age-related bias, and because of the low number of available studies. The final search was restricted to full-text articles that were written in English and published between 1990 and November 2018 in peer-reviewed journals.

### 2.3. Study Selection

To be included in the analysis, studies had to meet the following criteria: (1) be double-blind placebo-controlled randomized clinical trials, clinical trials, evaluation studies, and validation studies; (2) include adult participants with RA, SpA, or PsA, respectively; (3) compare placebo with active treatments including non-steroidal anti-inflammatory drugs (NSAIDs), biological disease-modifying anti-rheumatic drugs (bDMARDs), conventional synthetic DMARDs (csDMARDs), targeted synthetic DMARDs (tsDMARDs), and other immunosuppressant agents; (4) include at least 50 participants in each study group (placebo/active comparator); (5) report objectively measured disease-specific and clinically relevant primary endpoints (see Table S1). Studies without the required study design—e.g., meta-analysis, single-blind studies, long-term extension studies (open-label or without a placebo group), post hoc analyses—and duplicates were excluded. Especially, studies not meeting these primary endpoints, for example, with nonclinical and radiographic outcomes, or other parameters for quality of life, biomarkers, and pharmacokinetics as outcomes were excluded. Figure S1 summarizes the steps of the selection process.

### 2.4. Risk of Bias

A simple risk-of-bias assessment of individual studies was performed by two independent reviewers, with trials with low risk of bias being defined as fulfilling the three following criteria: (i) adequate concealment of allocation, (ii) inclusion of at least 50 patients per study group, and (iii) dropout rate less than 15%.

Additionally, the authors had no selection bias and were not supported by any of the pharmaceutical companies whose products were used in the included clinical studies.

### 2.5. Data Collection Process

A data extraction form was developed for data collection. One reviewer (K.R.) extracted and selected the data, whereas a second reviewer (M.S.) was consulted when necessary, and doubts were discussed to consensus. For the selected studies, the extracted data included: first author, name of trial, year and month of publication, study sites, number of patients in each study group (placebo/active comparator), drug class of the active treatment being investigated (NSAIDs, bDMARDs, csDMARDs, tsDMARDs, others), route of administration of the study drug (oral/parenteral), baseline characteristics of the placebo group (percentage of female participants, percentage of participants of Caucasian ethnicity, mean age, mean duration of symptoms/disease, concomitant and prior medication), results in clinical outcomes (depending on the specific disease), time point of outcome measurement (eventually more than one time point). The time point at which the primary endpoint was assessed was defined as the duration of the trial. Study sites were reported as the continent(s) in which the studies were conducted. For studies investigating more than one dosage of the active study drug, results in clinical outcomes were reported as the mean of the results of all the active comparator groups.

### 2.6. Data Synthesis and Analysis

Information on each publication was collected in an Excel sheet and then transferred into SPSS (IBM Corp. Released 2017. IBM SPSS Statistics for Macintosh, Version 25.0. Armonk, NY, USA: IBM Corp.) and R (R Core Team, 2021) for further analyses and comparisons.

Descriptive statistics were calculated using unweighted data. The Kolmogorov–Smirnov test, Chi-Quadrat test, and binomial test were performed to analyze normal distribution of variables describing baseline characteristics of the placebo groups. Not normal distributed variables were then compared using the Mann–Whitney-U test for independent samples, whereas normal distributed variables were compared using the t-test for independent samples.

Analyses of correlations and linear regressions between clinical endpoints and baseline characteristics of placebo groups were performed for studies conducted in the same disease after weighting of data according to the number of participants in the placebo groups.

The Pearson correlation coefficient and *p*-values were given for bivariate correlations, for significant correlations with *p* < 0.05 and |r| > 0.3. R^2^, and *p*-values for ANOVA and regression coefficients (beta coefficients: b0 = intercept; b1 = slope) were calculated using linear regressions.

### 2.7. Sample Size Calculations

Sample size calculations were performed with the SAS system (SAS Institute Inc., Cary, NC, USA, 2013), using the POWER procedure Fisher’s exact conditional test for two proportions, with alpha of 0.05 and a nominal statistical power of 0.9 which were used to calculate the different sample sizes as indicated for exemplary randomized placebo-controlled phase III trials, when results of response rates in phase I/II studies were available.

### 2.8. Role of the Funding Source

For publication fees only.

## 3. Results

### 3.1. Search Result and Characteristics of Eligible Studies

The systematic literature search resulted in the identification of 2137 records. A total of 152 publications met all of the eligibility criteria and were included into the review and in further analysis. A flowchart detailing the process of study identification and selection is shown in Figure S1. All of the eligible studies comprised a placebo intervention, as well as an active comparator group. Out of these, 110 studies were conducted in patients with RA (NSAIDs, *n* = 9; csDMARDs, *n* = 8; bDMARDs, *n* = 69; tsDMARDs, *n* = 13; others, *n* = 11), 18 studies were conducted in patients with SpA (NSAIDs, *n* = 3; bDMARDs, *n* = 14; tsDMARDs, *n* = 1), and 24 studies were conducted in patients with PsA (csDMARDs, *n* = 2; bDMARDs, *n* = 21; tsDMARDs, *n* = 1). Most treatments were administered either subcutaneously or intravenously (65%) which reflects the fact that bDMARDS were the most frequently investigated active treatments. Concomitant therapy with csDMARDs was applied in most studies (70.1%) for which this type of information was provided (135 out of 152). A detailed list of the characteristics of included studies is provided in Table S2.

### 3.2. Placebo Study Populations in Chronic Arthritic Diseases

The selected studies included a total of 21,616 participants in the placebo groups (RA, *n* = 16,945; PsA, *n* = 2872; SpA, *n* = 1799), without a significant difference regarding the number of participants per placebo group in the three different chronic arthritic diseases (RA, *n* = 147.4 ± 104.4; PsA, *n* = 119.7 ± 53.1; SpA, *n* = 99.9 ± 29.3). A comparison of study participants’ characteristics and of the study durations is shown in Table 1. The mean percentage of females differed significantly between the three assessed chronic arthritic diseases and ranged from 38.2% in SpA, over 49.0% in PsA, to 79.0% in RA. Similarly, the mean age ranged from 38.2 years in SpA, over 48.7 years in PsA, to 52.8 years in RA. The included studies investigated patients with chronic arthritic diseases with mean duration of disease ranging from 3.2 years in SpA, over 7.9 years in PsA, to 8.0 years in RA, and mean duration of symptoms ranging from 8.3 years in SpA to 16.4 years in PsA (not reported for RA). The mean study duration ranged from 16.4 weeks in SpA, to 17.9 weeks in PsA, to 24.6 weeks in RA. No significant correlations were found between RA, PsA, or SpA and nominal variables describing locations of the included clinical trials, the type of active treatment being investigated, the route of administration, and permitted concomitant medication.

### 3.3. Placebo Effects in Clinical Trials Investigating Chronic Arthritic Diseases

To investigate possible placebo effects in clinical trials conducted in chronic arthritic diseases, this study aimed in identifying any strong significant correlations between baseline characteristics of the placebo groups and clinically relevant endpoints. Therefore, analyses of bivariate correlations and linear regressions were performed for studies conducted in the same disease after weighing of data according to the number of participants in the respective placebo groups. The statistical measure, the coefficient of determination (R^2^), represents the proportion of variance for a dependent variable that is explained by an independent input variable [10]. A strong correlation can be anticipated when R^2^ is above 0.8, indicating that 80% of the variation in the output can be explained by the input variable.

**Figure 1:**
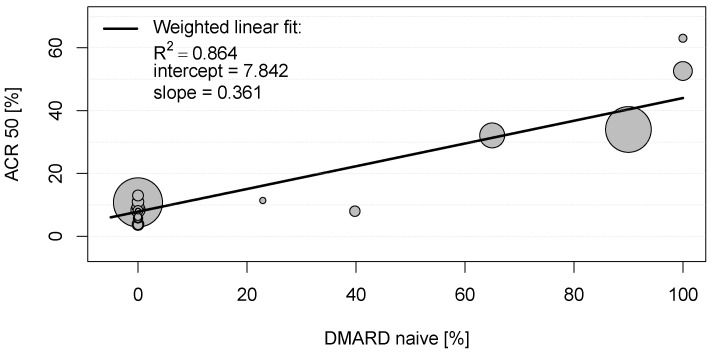
Scatter plot showing the positive weighted Pearson correlation between proportions of patients with rheumatoid arthritis achieving American College of Rheumatology score 50 (ACR50) and proportions of disease-modifying anti-rheumatic drug (DMARD)-naïve patients in the placebo groups. Each circle represents the study size for an individual placebo group.

In the eligible clinical studies for rheumatoid arthritis, the American College of Rheumatology scores (ARC20, ACR50, and ACR70, respectively) were the most frequently used primary endpoints (see Table S2) and thus used for the bivariate correlation analysis. As can be seen in Table S3, several mild to moderate significant correlations between these clinical endpoints and baseline characteristics of placebo groups could be observed. However, a strong positive correlation was detected between the proportion of patients achieving ACR20, ACR50, and ACR70 in the placebo groups and DMARD-naïvety (0.899 < |r| < 0.930; each with *p* < 0.001; Table S3). To explore this relationship, a scatter plot was constructed for the proportions of patients with RA achieving ACR50 and proportions of DMARD-naïve patients in the placebo groups (see Figure 1). The weighted linear fit revealed an R^2^ of 0.864 with a *p*-value < 0.001, again indicating a strong statistically significant positive correlation between ACR50 and DMARD-naïvety in RA patients.

**Figure 2:**
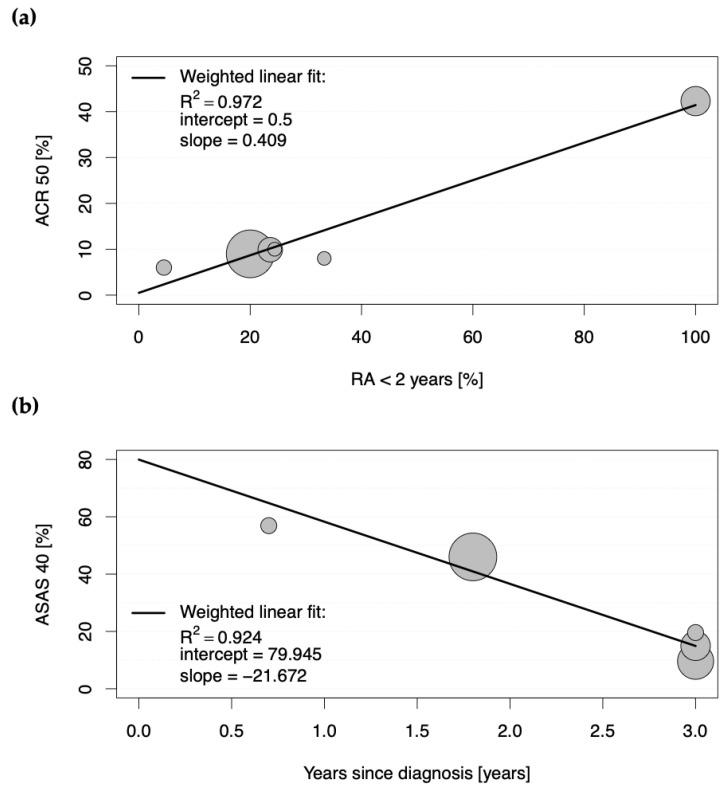
Scatter plots showing the positive weighted Pearson correlation between proportions of patients with (**a**) rheumatoid arthritis (RA) achieving American College of Rheumatology score 50 (ACR50) and proportions of patients with less than a two-year duration of RA in the placebo groups, and with (**b**) axial spondyloarthritis achieving Assessment of Spondyloarthritis International Society 40 (ASAS40) response in the placebo groups of eligible studies and duration of years since diagnosis. Each circle represents the study size for an individual placebo group.

The second determinant of a strong placebo effect was an early disease stage (RA < 2 years), as a strong positive correlation was observed between the proportions of patients achieving ACR50 and ACR70 in the placebo groups and the proportions of patients with early rheumatoid arthritis (0.985 < |r| < 0.986; each with *p* < 0.001; Table S3). Figure 2a depicts a scatter plot for the positive weighted Pearson correlation between those patients with early disease stage and the achievement of ACR50 in the placebo groups, with the weighted linear fit revealing an R^2^ of 0.972 (*p* < 0.001).

These results suggest that 86.4% and 97.2% of the ACR50 scores in the placebo groups in clinical trials for RA are explained by DMARD-naïvety and an early stage of the disease, respectively.

As true for RA, ACR20, ACR50, and ACR70 response scores were also the most frequently assessed primary endpoints in clinical studies for PsA (see Table S2) and thus also used here for the bivariate correlation analysis. All significant correlations with |r| > 0.300 and *p* < 0.001 between baseline characteristics of the placebo groups and proportions of patients achieving the mentioned ACR scores in the placebo groups are shown in detail in Table S4. The strongest correlations were detected between the proportions of patients achieving ACR70 in the placebo groups and concomitant (|r| < 0.964, *p* < 0.001) and prior DMARD therapy (|r| < −0.905, each *p* < 0.001), respectively. These results indicate that concomitant therapy with DMARDs favor greater responses in the placebo groups, whereas prior DMARD therapy seems to have a negative influence on clinical outcomes in the placebo groups. However, since this counts only for a small proportion of patients achieving ACR70 (around 3%) and since the analysis included only four eligible studies, we do not want to overemphasize those data.

For studies conducted in SpA, the Assessment of Spondyloarthritis International Society (ASAS) response criteria (ASAS20, ASAS40, ASAS5/6, and ASAS partial remission) were the most frequently assessed primary endpoints (see Table S2). All significant correlations with |r| > 0.300 and *p* < 0.001 between baseline characteristics of the placebo groups and proportions of patients achieving the mentioned ASAS response scores in the placebo groups are shown in detail in Table S5. Here, the proportions of patients achieving ASAS20, ASAS40, ASAS5/6, and ASAS partial remission criteria positively correlated with corresponding proportions of patients achieving those response criteria in the active comparator groups (0.409 < |r| < 0.930; each with *p* < 0.001; Table S5). However, the strongest correlation was observed as negative between the proportions of patients achieving the single ASAS response criteria and the duration of the disease since diagnosis (−0.772 < |r| < −0.961; each with *p* < 0.001; Table S3). To analyze this relationship in more detail, a scatter plot was constructed for the proportions of patients with SpA achieving ASAS40 in the placebo groups and duration of disease since diagnosis (see Figure 2b). The weighted linear fit revealed an R^2^ of 0.924 with a *p*-value < 0.001, confirming a strong positive, statistically significant correlation.

## 4. Discussion

Although the amount of literature on placebo effects in various diseases is rising, this is the first study to systematically analyze the placebo effect on disease activity in the clinical context of several chronic arthritic diseases. There are multiple disease-modifying agents for these diseases and a range of outcomes that include subjective (e.g., assessment of disease activity and pain) and objective measurements (e.g., laboratory and radiographic biomarkers). To assess the evidence for the effects of new treatments, the treatment approaches have to be tested formally in double-blinded randomized placebo controlled trials. This provides an excellent opportunity to explore the placebo effect and its determinants in chronic arthritic diseases.

This analysis examined the influence of placebo effects in treatment studies with placebo and active comparator groups of patients with RA, PsA, and axial SpA. The literature search yielded 152 eligible studies, with a total of 21.616 participants included in the placebo groups. To identify determinants of higher response in placebo groups, this study aimed in identifying any strong significant correlation between baseline characteristics of the placebo groups and clinically relevant endpoints. Analyses of bivariate correlations and linear regressions revealed two major determinants of the placebo effect in chronic arthritic diseases, namely, (i) DMARD naïvety and (ii) early stage of disease.

In clinical studies conducted in RA, placebo group participants, who have been completely DMARD-naïve before entry into the clinical trial, showed an ACR20 score of 73.0%, an ACR50 of 57.8%, and an ACR70 of 45.0% (compared to ACR20 of 85.5%, ACR50 of 70.9%, and ACR70 of 65.0% in the active comparator groups, respectively). This is quite impressive, considering that ACR50 implicates an improvement of symptoms of more than 50%. In contrast, placebo group participants who were previously treated with DMARDs (DMARD-naïvety of 0%) showed an ACR20 of 21.3%, an ACR 50 of 7.8%, and an ACR70 of only 2.4% (compared to ACR20 of 50.8%, ACR50 of 26.5%, and ACR70 of 11.5% in the active comparator group). Unfortunately, the information on proportions of DMARD-naïve study participants was not given in the included studies conducted in SpA and PsA. However, any prior bDMARD treatment of patients with PsA correlated negatively with ACR70 scores in the placebo groups, again indicating that the absence of prior experience with treatments is associated with more neutral expectations towards treatment response, thus critically determining the magnitude of placebo effect.

The second determinant of increased response in placebo groups of studies conducted in chronic arthritic diseases is an early stage of disease. Whereas only a moderate positive correlation could be observed for placebo group participants in RA achieving high ACR20 scores (R^2^ of 0.713), a strong positive correlation was observed between proportions of patients achieving significantly relevant ACR50 and ACR70 responses (R^2^ of 0.972 and 0.970, respectively) when the disease duration for all study participants was less than two years. Here, an ACR50 of 42.3% and an ACR70 of 27.3% could be achieved in the placebo groups, whereas the active comparator groups yielded 57.4% and 42.6%, respectively. When only around 20% of patients had an early disease stage, the percentages declined to 9.6 for ACR50 and 2.9 for ACR70 in the placebo groups (versus 28.7 and 13.5 in the active comparator groups, respectively). Accordingly, longer disease duration was associated with significantly lower ASAS response criteria in the placebo groups of clinical studies conducted in SpA. A meta-analysis of randomized controlled trials in fibromyalgia also observed lower placebo effect sizes in trials of participants with longer mean disease duration and thus consistently came to the conclusion that early intervention in fibromyalgia is more likely to give a good outcome [11]. Conclusively, these results reflect the importance of early treatment, which significantly increases the chances of achieving good clinical outcomes. In fact, the longer a patient is affected by a disease, the more complicated his disease evolves; a patient’s expectancies may decrease, and as a result, it becomes harder to improve outcomes by either active treatment, placebo, or any other factors that influence contextual response.

**Figure 3:**
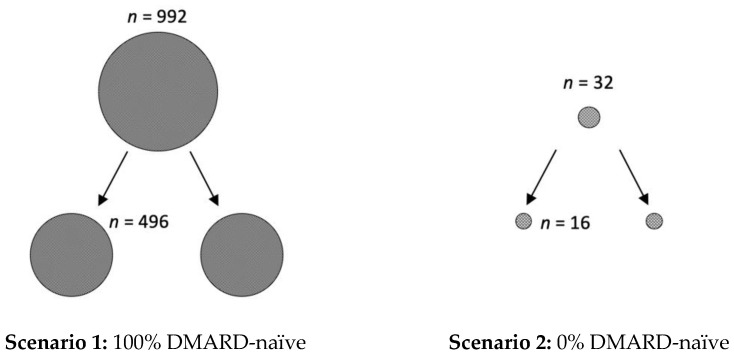
Impact of placebo effect on study size calculations. If phase I/II studies conducted in rheumatoid arthritis suggest a response rate of 70% for a new active drug, there are two feasible scenarios for study size calculations for phase III clinical trials. In scenario 1, all included patients are DMARD-naïve (100%) and the expected ACR50 response, due to the large placebo effect, is 60%. Here, the calculated number (*n*) of study participants is in total 992, with 496 for each of the two study arms. In scenario 2, all patients are pre-treated with DMARDs (0% DMARD naïve) and thus an ACR50 response score of 10% can be anticipated. Here, only 32 study participants, with 16 per arm, are required. Calculations were performed with the SAS system, using the POWER procedure Fisher’s exact conditional test for two proportions, with alpha of 0.05 and a nominal statistical power of 0.9.

However, in addition to the clinical relevance of the presented data, it is also important to create awareness of the underestimated impact of the placebo effect on study size calculations. For example, if phase I/II studies conducted in RA suggest a response rate of 70% for a new active drug, the calculated number of patients for each arm of the randomized placebo-controlled trial for phase III is 496, if 100% of patients are DMARD-naïve (with an expected ACR50 response of 60% in the placebo arm) (see Figure 3). However, the calculated number of required study participants for each arm of the trial is only 16, if all RA patients are pre-treated with a DMARD (0% DMARD-naïve; with an expected ACR50 response of 10% in the placebo arm). Similarly, 140 patients per study arm are needed when the patients are diagnosed with RA in less than two years (with an expected ACR50 response of 40%, and 60% in the active comparator group), compared to only 21, when the diagnosis was already longer ago (expected ACR50 of 10%).

Furthermore, not only is there a crucial difference in sample size for the sponsor who is conducting the clinical trial, but large placebo responses in such trials may also keep effective medication from reaching the market. In this context, it might be necessary to shift from more patient-reported to objective outcome measurements. However, a recently performed meta-analysis revealed that both objective and subjective outcome measures in the placebo arms of RA trials improved to a clinically meaningful extent, at least within the five clinical trials included in the analysis. The authors of this meta-analysis therefore came to the conclusion that the observed placebo responses may be more than just a psychological phenomenon [12]. Now, it is becoming essential that we improve our understanding of the underlying mechanisms.

### Limitations

Although the systematic literature search identified a large number of eligible studies, there are several limitations to this analysis. First, it is well known that there is a publication bias against negative results. Second, this analysis used only collective and not individual patients’ data of the placebo groups. Therefore, it was not possible to investigate any correlations between individual patient’s characteristics, including burden of disease, expectations, beliefs, anticipations of clinical improvements, and attitude towards the therapy and the medical staff with the clinical outcome. These parameters are well known determinants of the placebo effect [13]. Additionally, as studies included in the analysis were heterogeneous, conducted under different conditions and assessing different clinical outcomes, cross-disease comparisons were limited to variables, which were available for all studies. Additionally, due to this reason, it was not possible to conduct a meaningful multivariate analysis. Furthermore, only 7.11% of all studies identified by the applied search strategy were considered eligible for further analysis. The selection of only studies addressing clinical outcomes as a primary endpoint was driven by previous findings of placebos being ineffective for almost all objective outcomes (e.g., radiographic progression) [14]. The analysis was restricted to studies with at least 50 participants per group, which was due to the facts that the sample size (i) significantly reduces the risk of bias and (ii) is a major determinant of placebo effects in osteoarthritis [14]. Only studies published after 1990 were included since they were considered as more reliable to fulfill current standards of clinical trials’ design and therefore be more adequate for comparisons. A further limitation of this study is the lack of three-armed trials, including a non-treatment control group. Although essential to distinguish improvements in the placebo group from phenomena such as spontaneous remission or regression to the mean, which are often mistakenly understood as placebo effect [15], a trial arm without any treatment has to be considered as unethical [16].

## 5. Conclusions

In conclusion, this study has demonstrated that an early stage of chronic arthritic disease and lack of previous treatment favor a significantly higher response rate in placebo groups of double-blinded randomized controlled clinical trials. In addition to the clinical relevance, this work allows important conclusions for the design of clinical trials in chronic arthritic diseases, and may have an impact also for other diseases where a large placebo effect size is anticipated.

## Figures and Tables

**Table 1:** Comparison of characteristics of participants in placebo (PBO) groups and duration of studies conducted in rheumatoid arthritis (RA), psoriatic arthritis (PsA), and spondyloarthritis (SpA).

	RA	PsA	SpA	*p*	*p*	*p*
	(Mean ± SD)	(Mean ± SD)	(Mean ± SD)	RA vs. PsA	PsA vs. SpA	RA vs. SpA
Participants per PBO group (*n*)	147.4 ± 104.4	119.7 ± 53.1	99.9 ± 29.3	0.595 n.s.	0.274 n.s.	0.167 n.s.
Female (%)	79.0 ± 9.5	49.0 ± 9.7	34.1 ± 16.0	<0.001 ***	0.001 **	<0.001 ***
Mean age (years)	52.8 ± 2.5	48.7 ± 2.1	38.2 ± 5.1	<0.001 ***	<0.001 ***	<0.001 ***
Duration of disease (years)	8.0 ± 3.1	7.9 ± 2.2	3.2 ± 1.9	0.332 n.s.	<0.001 ***	<0.001 ***
Duration of symptoms (years)	n.r.	16.4 ± 2.5	8.3 ± 4.0	-	<0.001 ***	-
Study duration (weeks)	24.6 ± 19.1	17.9 ± 6.5	16.4 ± 13.9	0.252 n.s.	0.388 n.s.	0.007 **

** *p* < 0.01, *** *p* < 0.001, n.s., not significant. Data were analyzed using Mann–Whitney U test for independent samples and are expressed as mean ± standard deviation (SD). Other abbreviations: *n*, number; n.r., not reported.

## Data Availability

Not applicable, as data are provided in the Supplementary files.

## Supplementary Materials

The following supporting information can be downloaded at: https://www.mdpi.com/article/10.3390/jcm12020429/s1, Figure S1: Flowchart detailing the process of study identification and selection from the literature; Table S1: List of double-blind placebo-controlled randomized clinical trials in RA, SpA, or PsA included in the analysis; Table S2: List of the characteristics of included studies; Table S3: Results of bivariate correlation analyses from clinical endpoints and baseline characteristics of placebo groups; Table S4: Correlations with |r| > 0.300 and *p* < 0.001 between baseline characteristics and proportions of patients achieving ACR scores in RA-placebo group; Table S5: Correlations with |r| > 0.300 and *p* < 0.001 between baseline characteristics and proportions of patients achieving the ASAS response scores in the SpA-placebo groups.
