# Supplementary material for: Clinical Trials in Chronic Arthritic Diseases with Underestimated Impact of Placebo Effects on Study Size Calculation
Source: J Clin Med. 2023 Jan 5;12(2):429. doi: 10.3390/jcm12020429 (PMC9863871; doi:10.3390/jcm12020429)
Supplement: Supplementary file 1 [file jcm-12-00429-s001.zip › jcm-2089559-supplementary.pdf]

Journal of
Clinical Medicine

MDPI

Supplementary

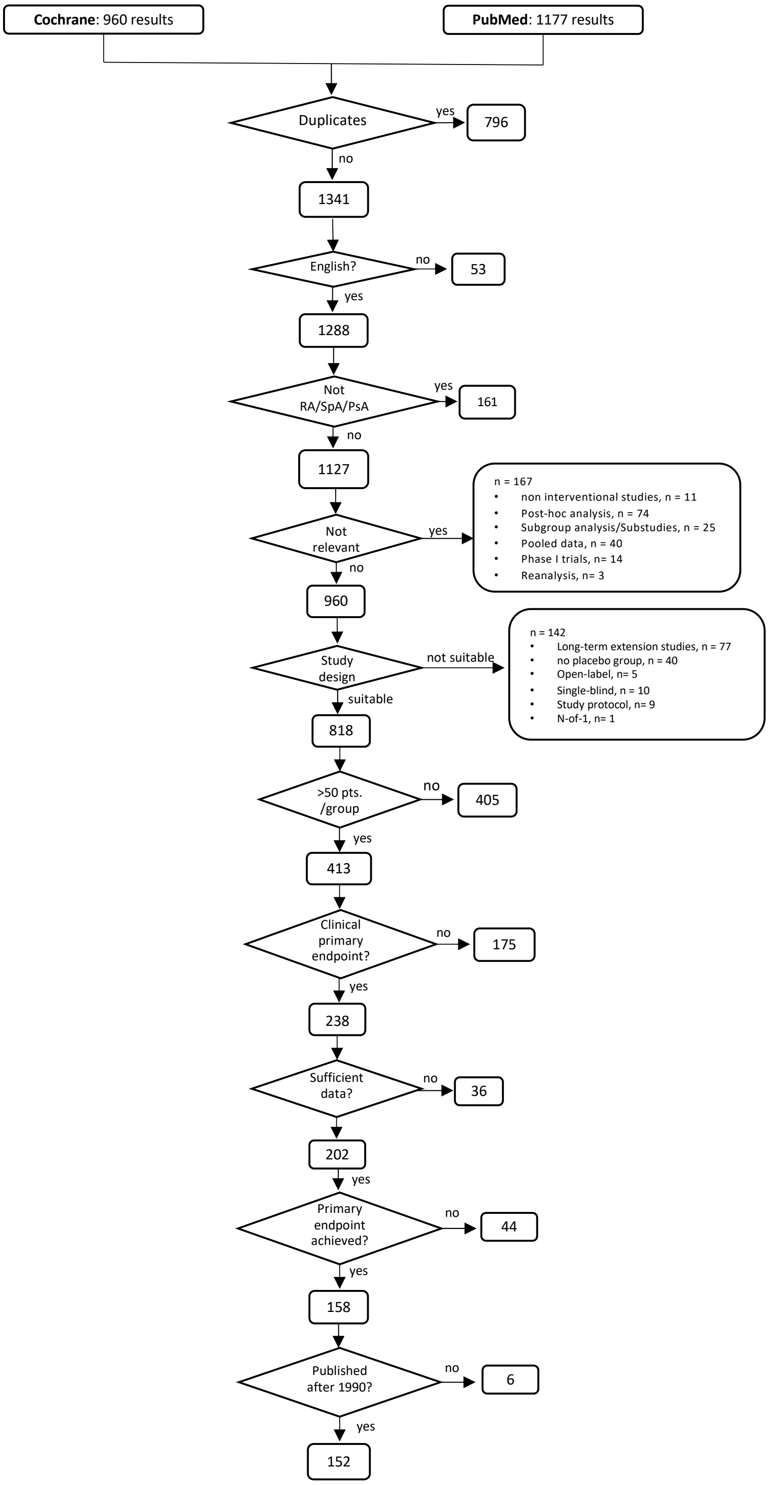

Figure S1. Flow-chart describing the main steps in the selection process of publications from the systematic literature search to the publications considered eligible for further analysis.

Table S1. List of clinical primary endpoints used for the selection of eligible studies conducted in rheumatoid arthritis (RA), psoriatic arthritis (PsA) and axial spondyloarthritis (SpA).

Disease	Clinical primary endpoints
**Rheumatoid arthritis (RA)**	ACR20 / ACR50 / ACR70 = American College of Rheumatology response scores<br>CDAI = clinical disease activity index<br>DAS = disease activity score (referred to 44 joints)<br>DAS28 = disease activity score (referred to 28 joints)<br>DAS28-CRP = DAS28 with C-reactive protein<br>DAS28-ESR = DAS28 with Erythrocyte Sedimentation Rate<br>Pain on VAS (1-10 cm)<br>Remission (Pinal criteria)<br>Ritchie Articular Index
**Psoriatic arthritis (PsA)**	ACR20 / ACR50 /ACR70<br>PASI = Psoriasis Area and Severity Index<br>PSARC = Psoriatic Arthritis Response Criteria
**Spondyloarthritis (SpA)**	ASAS20 / ASAS40 /ASAS5/6 / ASAS70 = Assessment of SpA international Society response criteria<br>BASDAI = Bath's Ankylosing Spondylitis Disease Activity Index<br>BASFI = Bath's Ankylosing Spondylitis Functional Index<br>BAS-G = Bath Ankylosing Spondylitis Patient Global Score<br>MASES = Maastricht Ankylosing Spondylitis Enthesitis Score<br>Pain on VAS (1-10 cm)<br>PSpARC = Peripheral Spondyloarthritis Response Criteria

Table S2. List of double-blind placebo-controlled randomized clinical trials, clinical trials, evaluation studies and validation studies conducted in rheumatoid arthritis (RA), psoriatic arthritis (PsA) and spondyloarhtritis (SpA) retrieved after systemic literature search and selection process grouped by type of active treatment. Abbreviations: Drug Class: bDMARDs, biological disease-modifying anti-rheumatic drugs; csDMARDs, conventional synthetic disease-modifying anti-rheumatic drugs; NSAIDs, nonsteroidal anti-inflammatory drugs; tsDMARDs, targeted synthetic disease-modifying anti-rheumatic drugs. Application: iv, intravenous; po, perioral; sc, subcutaneous. Primary endpoint: ACR, American College of Rheumatology; ASAS, Assessment of SpondyloArthritis international Society; ASDAS, Ankylosing Spondylitis Diseases Activity Score; BASDAI, Bath Ankylosing Spondylitis Disease Activity Index; BASFI, Bath Ankylosing Spondylitis Functional Index; CDAI, Clinical Disease Activity Index; CRP, C-reactive Protein; DAS, Disease Activity Score; ESR, Erythrocyte Sedimentation Rate; HAQ-DI, Health Assessment Questionnaire Disability Index; PASI, Psoriasis Area and Severity Index; PGA, Physician's Global Assessment; PsARC, Psoriatic Arthritis Response Criteria; PtGA, Patient's Global Assessment; SF-36, Short-Form 36 health questionnaire; SJC, Swollen Joint Count; sLDA, sustained low disease activity; sREM, sustained remission; TJC, Tender Joint Count; VAS, Visual Analog Scales.

**RA**							
**Drug class**	**Reference**	**Active Compound**	**Application (po, sc, iv)**	**Phase II/III**	**Duration (weeks)**	**Primary endpoint**	**Publication Year (Month)**
NSAIDs							
	Simon	Celecoxib	po	III	12	PtGA, PGA, Pain (VAS)	1999 (Nov)
	Collantes	Etoricoxib	po	III	12	PtGA, PGA, TJC, SJC	2002 (May)
	Furst	Meloxicam	po	III	12	SJC, TJC , PtGA, PGA, pain	2002 (Mar)
	Matsumoto	Etoricoxib	po	III	12	PtGA, PGA, TJC, SJC	2002 (Aug)
	Williams	Valdecoxib	po	III	12	ACR20, TJC, PJC, SJC, PtGA, PGA	2006 (Feb)
	Greenwald	Etoricoxib	po	III	12	ACR20	2011 (Oct)
	Bickham	Etoricoxib	po	III	12	DAS28-CRP	2016 (Aug)
	Gibofsky	Valdecoxib	po	II	12	ACR20	2007 (Jun)
	Geusens	Lumiracoxib	po	II	26	ACR20	2004 (Nov)
csDMARDs							
	Strand	Leflunomide	po	II	52	ACR20	1999 (Nov)
	Smolen	Leflunomide	po	III	24	TJC, SJC, PtGA, PGA	1999 (Jan)
	Mladenovic	Leflunomide	po	II	24	TJC, SJC, TJS, SJS	1995 (Nov)
	Kremer	Leflunomide	po	II	24	ACR20	2002 (Nov)
	HERA Study Group	Hydroxychloroquine	po	II	36	Pain (VAS), PtGA, PGA, grip strength	1995 (Feb)
	Edwards	Methotrexate	po	III	60	DAS	2018 (Jan)
	O'Dell	MTX, SSZ, HCQ	po	III	104	ACR20	2002 (May)
	Capell (MASCOT)	MTX, SSZ	po	III	24	DAS	2007 (Feb)
bDMARDs							
	Nishimoto	MRA (anti-IL-6-AB)	iv	II	12	ACR20	2004 (Jun)
	Rigby (STAGE)	Ocrelizumab	iv	III	48	ACR20	2012 (Feb)
	Furst (DOSEFLEX)	Certolizumab pegol	sc	III	34	ACR20	2015 (Feb)
	Weinblatt (REALISTIC)	Certolizumab pegol	sc	III	12	ACR20	2012 (Dec)
	Smolen (CERTAIN)	Certolizumab pegol	sc	III	24	CDAI	2015 (May)
	Keystone	Adalimumab	sc	III	52	ACR20	2004 (May)
	Burmester (FUNCTION)	Tocilizumab	iv	III	52	DAS28-ESR	2016 (Jun)
	Smolen (OPTION)	Tocilizumab	iv	III	24	ACR20	2008 (Mar)
	Cohen (REFLEX)	Rituximab	iv	III	24	ACR20	2006 (Sep)
	Kremer	Abatacept	iv	III	52	ACR20	2006 (Jun)
	Keystone	Etanercept	sc	II	16	ACR20	2004 (Feb)
bDMARDs							
	Schiff (ATTEST)	Abatacept / Infliximab	iv	III	48	DAS28-ESR	2008 (Aug)
	Maini	Infliximab	iv	III	30	ACR20	1999 (Dec)
	Smolen (PRESERVE)	Etanercept	sc	III	88	DAS28 < 3.2	2013 (Mar)
	Moreland	Etanercept	sc	III	24	ACR20, ACR50	1999 (Mar)
	Blanco	Secukinumab	sc	III	52	ACR20	2017 (Jun)
	Weinblatt (ARMADA)	Adalimumab	sc	II	24	ACR20	2003 (Jan)
	Genovese	Abatacept	iv	III	24	ACR20	2005 (Sep)
	Burmester	Mavrilimumab	sc	II	24	DAS28-CRP, ACR20	2017 (Jun)

	Smolen	Certolizumab pegol	sc	III	24	ACR20	2009 (Jun)
	Yazici (ROSE)	Tocilizumab	sc	III	24	ACR50	2012 (Feb)
	St Clair	Infliximab	iv	III	54	ACR-n	2004 (Nov)
	Weinblatt	Fostamatinib	sc	III	52	ACR20	2014 (Dec)
	Genovese	Decernotinib	sc	II	24	ACR20, DAS28-CRP	2016 (Jan)
	Cohen	Anakinra	sc	II	24	ACR20	2004 (Sep)
	Emery (SERENE)	Rituximab	iv	III	48	ACR20	2010 (Sep)
	Emery	Rituximab	iv	II	24	ACR20	2006 (May)
	Mease (SUNRISE)	Rituximab	iv	II	48	ACR20, DAS28	2010 (May)
	Cohen	Anakinra	sc	II	24	ACR20	2002 (Mar)
	Takeuchi (SIRROUND-D)	Sirukumab	sc	III	52	ACR20	2017 (Dec)
	Genovese	Fostamatinib	sc	III	24	ACR20	2014 (Nov)
	Horslev-Petersen (OPERA)	Adalimumab	sc	III	48	DAS28-CRP < 3.2	2014 (Apr)
	Stohl	Belimumab	iv	II	48	ACR20	2013 (May)
	Bresnihan	IL-1-R-Antagonist	sc	II	24	ACR criteria	1998 (Dec)
	Alten	Canakinumab	sc, iv	II	12	ACR50	2011 (Jul)
	Genovese (MOBILITY)	Sarilumab	sc	III	52	ACR20	2015 (Jun)
	Smolen (GO-AFTER)	Golimumab	sc	III	84	ACR20	2009 (Jul)
	Ostergaard	Ofatumumab	iv	II	24	ACR20, DAS28, EULAR	2010 (Aug)
	Weinblatt	Clazakizumab	sc	II	24	ACR20	2015 (Oct)
	Genovese (TOWARD)	Tocilizumab	iv	III	24	ACR20 (24W)	2008 (Oct)
	Nishimoto (SATORI)	Tocilizumab	iv	III	24	ACR criteria, DAS-28	2009
	Damjanov	SBI-087	sc	II	24	ACR20	2016 (Dec)
	Emery (RADIATE)	Tocilizumab	iv	III	24	ACR20	2008 (Nov)
	Kivitz (BREVACTA)	Tocilizumab	iv	III	24	ACR20	2014 (Nov)
bDMARDs							
	Emery	Certolizumab pegol	sc	III	52	sREM, sLDA	2017 (Jan)
	Weinblatt	Certolizumab pegol	sc	III	52	DAS28-ESR < 3.2	2017 (Oct)
	Kavanaugh (OPTIMA)	Adalimumab	sc	III	26	DAS28-CRP < 3,2	2013 (Jan)
	Aletaha	Sirukumab	sc	III	52	ACR20	2017 (Mar)
	Yamamoto (J-RAPID)	Certolizumab pegol	sc	III	24	ACR20	2014 (Sep)
	Yamamoto (HIKARI)	Certolizumab pegol	sc	III	24	ACR20	2014 (Jul)
	Weinblatt (GO-FURTHER)	Golimumab	sc	III	24	ACR20	2013 (Mar)
	Takeuchi (GO-MONO)	Golimumab	sc	II/III	24	ACR20	2013 (Sep)
	Kremer	Abatacept	iv	II	48	ACR20	2005 (Aug)
	Kim	Infliximab	iv	III	30	ACR20	2013 (Dec)
	Keystone	CZP	sc	III	52	ACR20	2008 (Nov)
	Huizinga	Sarilumab	sc	II	12	ACR20	2014 (Sep)
	Taylor	Ofatumumab	iv	III	24	ACR20	2011 (Dec)
	Miyasaka (CHANGE)	Adalimumab	sc	II/III	24	ACR20	2008
	Tanaka (GO-FURTH)	Golimumab	sc	II/III	16	ACR20	2012 (Jun)
	Fleischmann (FAST4WARD)	CZP	sc	III	24	ACR20	2009 (Jun)
	Li	Golimumab	sc	III	56	ACR20	2016 (Nov)
	van de Putte	Adalimumab	sc	III	26	ACR20	2004 (May)
	Fan	Leining	iv	II	24	ACR20	2014 (Nov)
	Pavelka	Etanercept	sc	III	52	DAS28 < 3.2	2017 (Sep)
	Furst	Pegsunercept	sc	II	12	ACR20	2005 (Dec)
	Chen	Etanercept	sc	III	24	ACR20	2016 (Sep)
	Keystone (GO-FORWARD)	Golimumab	sc	III	24	ACR20	2009 (Jun)
	Westhovens	Abatacept	iv	III	52	DAS28-CRP < 2.6	2009 (Dec)
	van de Putte	Adalimumab	sc	II	12	ACR20	2003 (Dec)
**tsDMARDs**							
	Burmester	Tofacitinib	po	III	24	ACR20, HAQ-DI, DAS	2013 (Feb)
	Kremer	Tofacitinib	po	II	24	ACR20	2012 (Apr)
	Tanaka	Tofacitinib	po	II	12	ACR20	2015 (Jul)
	van der Heijde	Tofacitinib	po	III	104	ACR20	2013 (Mar)
	Kremer	Tofacitinib	po	II	52	ACR20	2013 (Aug)
	Kremer	Upadacitinib	po	II	12	ACR20	2016 (Dec)
tsDMARDs							
	Takeuchi	Peficitinib	po	II	12	ACR20	2016 (Jun)

	Genovese	Peficitinib	po	II	12	ACR20	2017 (May)
	Kremer	Tofacitinib	po	II	12	ACR20	2009 (Jul)
	Westhovens (DARWIN 1)	Filgotinib	po	II	24	ACR20	2017 (Jun)
	Kavanaugh (DARWIN 2)	Filgotinib	po	II	24	ACR20	2017 (Jun)
	Kivitz	Peficitinib	po	II	12	ACR20	2017 (Apr)
	Taylor	Baricitinib	po	III	52	ACR20	2017 (Feb)
**others**							
	Kondo	Tacrolimus	po	II	16	ACR20	2004 (Feb)
	Bruyn	Everolimus	po	II	12	ACR20	2008 (Aug)
	Yocum	Tacrolimus	po	III	24	ACR20	2003 (Dec)
	Tugwell	Cyclosporine	po	II	48	TJC	1995 (Jul)
	Furst	Tacrolimus	po	II	24	ACR20, TJC, SJC	2002 (Aug)
	McCarey (TARA)	Atorvastatin	po	III	24	DAS28	2004 (Jun)
	Ogrendik	Ornidazole	po	II	12	ACR	2006 (Oct)
	Buttgereit (CAPRA-2)	Prednisone	po	III	12	ACR20	2013 (Feb)
	Ogrendik	Roxithromycin	po	II	24	ACR20	2011 (Sep)
	Liu	TLHS	po	II	8	ACR20	2016 (Jul)
	Hetland	Cyclosporine	po	III	52	ACR20	2006 (May)
csDMARDs							
	Kaltwasser	Leflunomide	po	II	24	PsARC	2004 (Jun)
	Clegg	Sulfasalazine	po	II	36	TJC, PJC, PtGA, PGA	1996 (Dec)
bDMARDs							
	McInnes	Secukinumab	sc	III	54	ACR20	2015 (Sep)
	Kavanaugh (PALACE-1)	Apremilast	po	III	24	ACR20	2014 (Jun)
	McInnes (PSUMMIT-1)	Ustekinumab	sc	III	24	ACR20	2013 (Aug)
	Kavanaugh	Golimumab	sc	III	20	ACR20, PASI, SF-36	2009 (Apr)
	Ritchlin	Ustekinumab	sc	III	40	ACR20	2014 (Jun)
	Mease (RAPID-PsA)	Certolizumab pegol	sc	III	24	ACR20	2014 (Jan)
	Gottlieb	Ustekinumab	sc	II	12	ACR20	2009 (Feb)
	Mease	Brodalumab	sc	II	12	ACR20	2014 (Jun)
	Antoni (IMPACT 2)	Infliximab	iv	III	24	ACR20	2005 (Aug)
	Mease	Adalimumab	sc	II	24	ACR20	2005 (Oct)
	Antoni (IMPACT)	Infliximab	iv	III	50	ACR20	2005 (Apr)
	Mease	Alefacept	iv	II	24	ACR20	2006 (May)
	Schett	Apremilast	po	II	28	ACR20	2012 (Oct)
	Mease	Etanercept	sc	II	24	ACR20	2004 (Jul)
	Nash (SPIRIT-P2)	Ixekizumab	sc	III	24	ACR20	2017 (Jun)
	Mease (SPIRIT-P1)	Ixekizumab	sc	III	24	ACR20	2017 (Jan)
	Kavanaugh (GO-VIBRANT)	Golimumab	sc	III	124	ACR20	2017 (Nov)
	Mease	Abatacept	sc	III	24	ACR20	2017 (Sep)
	Edwards (PALACE-3)	Apremilast	po	III	52	ACR20	2016 (Jun)
	Cutolo (PALACE-2)	Apremilast	po	III	52	ACR20	2016 (Sep)
	Nakagawa	Brodalumab		II	12	PASI	2016 (Jan)
tsDMARDs							
	Gladman	Tofacitinib	po	III	24	ACR20	2017 (Oct)
NSAIDs							
	Dougados	Celecoxib	po	III	6	Pain (VAS), BASFI	2001 (Jan)
	van der Heijde	Etoricoxib	po	II	52	Back pain (VAS), PtGA, BASFI	2005 (Apr)
	Barkhuizen	Celecoxib	po	II	12	Pain, PtGA, BASFI	2006 (Sep)
bDMARDs							
	Landewé (RAPID-axSpA)	Certolizumab pegol	sc	III	24	ASAS20	2014 (Jan)
	Inman	Golimumab	sc	III	24	ASAS20	2008 (Nov)
	Sieper (INFAST)	Infliximab	iv	III	28	ASAS	2014 (Jan)
	Dougados	Etanercept	sc	III	12	ASAS40	2014 (Aug)
	Davis	Etanercept	sc	II	24	ASAS20	2003 (Nov)
	van der Heijde (ASSERT)	Infliximab	iv	II	24	ASAS20	2005 (Feb)
	Sieper (ABILITY-1)	Adalimumab	sc	III	12	ASAS40	2013 (Jun)
	van der Heijde	Adalimumab	sc	II	24	ASAS20	2006
	Mease (ABILITY-2)	Adalimumab	sc	III	156	PsARC40	2015 (Apr)
	Sieper	Golimumab	sc	III	16	ASAS20	2015 (Oct)

	Revicki (ATLAS)	Adalimumab	sc	III	284	Back pain (VAS), BASDAI	2008 (Jul)
	Bao	Golimumab	sc	III	24	ASAS20	2014 (Sep)
	Huang	Adalimumab	sc	III	12	ASAS20	2014 (Mar)
	Landewé (ABILITY-3)	Adalimumab	sc	III	68	ASDAS ≥2·1	2018 (Jul)
tsDMARDs							
	van der Heijde	Tofacitinib	po	II	16	ASAS20	2017 (Aug)

Table S3. Significant correlations between clinical endpoints and baseline characteristics of placebo (PBO) groups of studies conducted in rheumatoid arthritis (RA), with $p > 0.001$ and $|r| > 0.3$. r, Pearson correlation index; n.s., not significant.

		Correlations		
		**ACR20 PBO (%)**	**ACR50 PBO (%)**	**ACR70 PBO (%)**
ACR20 active comparator (%)	r	0.682***	0.679***	0.569***
ACR50 active comparator (%)	r	0.775***	0.833***	0.737***
ACR70 active comparator (%)	r	0.647***	0.807***	0.811***
Mean age (years)	r	-0.423***	-0.451***	-0.378***
Disease duration (years)	r	-0.513***	-0.676***	-0.624***
RA < 2 years (%)	r	0.845***	0.986***	0.985***
Concomitant steroids (%)	r	n.s.	-0.379***	-0.323***
Concomitant MTX (%)	r	0.319***	n.s.	n.s.
Any concomitant DMARDs (%)	r	0.351***	0.626***	n.s.
Prior DMARDs (%)	r	-0.804***	-0.848***	-0.611***
DMARDs-naive (%)	r	0.899***	0.930***	0.922***
Prior MTX (%)	r	-0.712***	-0.626***	0.889***
Prior anti-TNF (%)	r	-0.436***	-0.430***	n.s.
Prior bDMARDs other than anti-TNF (%)	r	-0.304***	-0.358***	-0.364***
Study duration (weeks)	r	0.537***	0.695***	0.638***

Table S4. Significant correlations between clinical endpoints and baseline characteristics of placebo (PBO) groups of studies conducted in psoriatic arthritis (PsA), with $p > 0.001$ and $|r| > 0.3$. r, Pearson correlation index; n.s., not significant.

		**Correlations**		
		**ACR20 PBO (%)**	**ACR50 PBO (%)**	**ACR70 PBO (%)**
ACR20 active comparator (%)	r	0.304***	n.s.	n.s.
ACR50 active comparator (%)	r	n.s.	n.s.	n.s.
ACR70 active comparator (%)	r	0.322***	n.s.	n.s.
Participants in PBO group (n)	r	0.489***	n.s.	0.301***
Female (%)	r	0.507***	0.596***	0.537***
Caucasian (%)	r	0.427***	-0.315***	-0.427***
Duration of PsA (years)	r	-0.310***	n.s	n.s.
Duration of psoriasis (years)	r	-0.412***	n.s.	n.s.
Enthesitis (%)	r	0.341***	n.s.	n.s.
Dactylitis (%)	r	n.s.	-0.308***	n.s.
Concomitant NSAIDs (%)	r	n.s.	-0.363***	0.572***
Concomitant steroids (%)	r	0.512***	0.348***	0.430***
Concomitant MTX (%)	r	0.421***	0.464***	0.574***
Concomitant DMARDs other than MTX (%)	r	0.609***	0.862***	0.964***
Any concomitant DMARDs	r	n.s.	0.833***	0.869***
Prior DMARDs (%)	r	n.s.	-0.739***	-0.484***
Prior anti-TNF (%)	r	0.689***	0.552***	0.536***
Any bDMARDs prior (%)	r	-0.392***	n.s.	-0.905**
Study duration (weeks)	r	0.389***	n.s.	n.s.
Publication year	r	0.536***	0.503***	0.547***

Table S5. Significant correlations between clinical endpoints and baseline characteristics of placebo (PBO) groups of studies conducted in spondyloarthritis (SpA), with $p > 0.001$ and $|r| > 0.3$. r, Pearson correlation index; n.s., not significant.

		**Correlations**			
		**ASAS20 PBO (%)**	**ASAS40 PBO (%)**	**ASAS5/6 PBO (%)**	**ASAS part. rem. PBO (%)**
ASAS20 active comparator (%)	r	0.679***	0.685***	0.776***	0.790***
ASAS40 active comparator (%)	r	0.740***	0.879***	0.913***	0.929***
ASAS5/6 active comparator (%)	r	n.s.	0.409***	0.635***	0.431***
ASAS partial remission active comparator (%)	r	0.901****	0.930***	0.925***	0.923***
Participants in PBO group (n)	r	n.s.	n.s.	0.752***	n.s.
Female (%)	r	0.348***	n.s.	n.s.	n.s.
Mean age (years)	r	-0.510***	n.s	n.s	-0.448***
Caucasian (%)	r	n.s	0.446***	n.s	0.428***
Years since diagnosis	r	-0.772***	-0.961***	-0.961***	-0.961***
Years since symptoms	r	-0.665***	-0.430***	-0.564***	-0.730***
HLA-B27 positive (%)	r	0.333***	n.s	0.370***	n.s
Concomitant NSAIDs (%)	r	n.s	n.s	-0.465***	n.s
Any concomitant DMARDs	r	n.s	-0.535***	0.385***	-0.419***
Prior anti-TNF (%)	r	n.s	n.s	-0.355***	-0.332***
Study duration (weeks)	r	0.523***	0.799***	0.929***	0.728***
Publication year	r	0.646***	0.545***	0.587***	0.623***
